# Primary health care situations in remote rural villages of the Savannakhet province, Lao People’s Democratic Republic

**DOI:** 10.1186/s41182-022-00482-9

**Published:** 2022-11-28

**Authors:** Daisuke Nonaka, Nouhak Inthavong, Kenzo Takahashi, Ketmany Chanthakoumane, Yuko Toyama, Chanthaly Luangphaxay, Tiengkham Pongvongsa, Sengchanh Kounnavong

**Affiliations:** 1 Faculty of Medicine, University of the Ryukyus, Okinawa, Japan; 2 Lao Tropical and Public Health Institute, Ministry of Health, Vientiane, Lao People’s Democratic Republic; 3 Teikyo University Graduate School of Public Health, Tokyo, Japan; 4 Savannakhet Provincial Health Department, Ministry of Health, Savannakhet, Lao People’s Democratic Republic

**Keywords:** Primary health care, Community health workers, Diarrhea; malaria, Laos

## Abstract

**Background:**

To improve the health of the rural population in the Lao People’s Democratic Republic (Lao PDR), the government has emphasized a primary health care approach in the Health Sector Reform Strategy by 2025. The objective of the present study was to describe the health-related situations of remote rural villages of the Lao PDR to inform strategies for promoting primary health care in such villages.

**Methods:**

Ten remote rural villages were purposively selected from the catchment areas of two health centers in the Xepon district, Savannakhet province. The surveyors collected data by conducting a questionnaire-based interview with village health volunteers and by observing the village environment in 2018. The survey focused on village situations on the eight elements of primary health care (health education; food supply and nutrition; safe water and basic sanitation; maternal and child health care; immunization; prevention and control of locally endemic diseases; treatment of common diseases and injuries; and provision of essential drug).

**Results:**

The common health problems were diarrhea, followed by malaria, and cough. The identified possible risk factors for the health problems were not washing hands with soap, open defecation, not boiling drinking water, not exclusively breastfeeding, presence of animal feces on the village ground, absence of garbage management system, not using a bed net when sleeping in the forest, and exposure to indoor cooking and tobacco smoke. In many villages, villagers were not able to eat enough food and did not eat protein-rich food and vegetables daily.

**Conclusions:**

Potential risk factors for the reported common health problems were often prevalent in the study villages. Villagers can address most of these risk factors, as interventions to address such risk factors do not require a large financial input. There is a need for intersectoral actions between the health and other sectors to address food shortages and indoor air pollution due to indoor cooking using biomass fuel.

**Supplementary Information:**

The online version contains supplementary material available at 10.1186/s41182-022-00482-9.

## Background

The Lao People’s Democratic Republic (Lao PDR) is a lower middle-income country located in Southeast Asia [1]. Within the country, there is wide disparity in most health indicators between the urban and rural populations. For example, the under-five mortality rate (per 1000) is 24 in children residing in urban villages versus 62 in children residing in hard-to-reach villages [2]. This disparity has long been persistent [2, 3]. To further improve the health of the rural population, the Lao government has emphasized a primary health care approach in the Health Sector Reform Strategy by 2025 [4]. The strategy aims at improving access to health services in remote rural areas by implementing both the new program (i.e., Village Health Workers program) and existing programs (including the Model Healthy Village program and Maternal and Child Health program). One of the priority areas of the strategy is the health financing with improved coverage of health insurance. The country started the National Health Insurance scheme in 2016 and achieved the 94% coverage of the National Health Insurance in 2018 [5, 6]. The strategy recommends that relevant policies and plans be further developed to inform the planning and implementation of improved services [4]. The present study is expected to contribute to the development of relevant policies and plans.

Village health volunteers are critical to the implementation of primary health care in Lao PDR, as they bridge the health sector and their communities. Their expected roles include helping health workers with outreach activities, providing health education for villagers, referring patients to healthcare facilities, facilitating prenatal care clinics at healthcare facilities, and conducting community-based surveillance for vital events and malaria (only in malaria endemic villages). They are selected by village executives and are then trained by health professionals. However, many women are not eligible to be selected as village health volunteers due to their educational attainment and poor ability to read, write and do simple mathematical calculations [7].

Many studies have been conducted in the rural villages of the Lao PDR, almost all of which are disease-specific (e.g., malaria, tuberculosis, diarrhea, and helminthiasis) [8–11] or topic-specific (e.g., childbirth, vaccination, and nutrition status) [12–14] with the view of a selective primary health care approach. Very few studies have been conducted in the rural villages of the Lao PDR that have aimed to capture a comprehensive picture of health-related situations in rural villages with the view of a horizontal primary health care approach. Therefore, information is lacking on the multiple determinants of common health problems in the same villages, making it challenging to identify solutions that villagers can easily address using the locally available resources.

The objective of the present study was to describe the health-related situations of 10 remote rural villages, which are located far from a healthcare facility, to inform strategies for promoting primary health care in remote rural villages of the Lao PDR.

## Methods

### Study design

A cross-sectional study was conducted in the 10 villages in the Xepon district, Savannakhet province. Data were collected from a questionnaire-based interview survey among village health volunteers, and an observational survey on the environmental conditions of the villages, in May 2018. Prevalence of villagers, with certain knowledge or behavior on a particular health issue, was estimated by village health volunteers. Reported health problems can be considered as outcome, with primary health care situations can be considered as exposures/predictors. As the present study used data with a small sample size, no statistical analysis was performed.

### Study villages and population

Xepon is a remote, poor district approximately 500 km from the national capital of Vientiane. Xepon has a population of 58,000 people and a total of 88 villages in 2017. According to the Xepon District Health Office, most of the people are of ethnic minorities, specifically the Tri and Mangkong people, comprising 75% of the total district population. They have their own distinctive languages, have limited formal education, and live in mountainous and forested areas that are often far from healthcare facilities. The majority of the population consists of farmers engaged in rice farming.

The healthcare facilities in Xepon comprised one district hospital and 12 health centers. For this study, we purposively chose two health centers (i.e., the Donsavanh and Manchi health centers) because these are accessible by car. In addition, malaria, which is one of the most important health problems in the district, is commonly seen in the catchment area [9, 15]. Then, we purposively chose a total of 10 hard-to-reach villages that are not located along a main road: Five from the 42 villages in the Dongsavanh health center’s catchment area and five from the 12 villages in the Manchi health center’s catchment area.

The median distance between the villages and the health centers was 22.5 km (interquartile range: 15–25.5 km). The median time taken to reach the villages from the health centers by motorbike with/without boat riding was 105 min (interquartile range: 60–127.5 min). The median population size was 166.5 people (interquartile range: 92–440.5 people) (Table 1).

**Table 1:** Characteristics of the study villages

Village name	Health center	Population	Number of households	Distance between village and health center (km)	Time taken to reach (min)*
Palay	Dongsavanh	377	51	25	120
Sapan	Dongsavanh	157	27	30	180
La En	Dongsavanh	222	33	27	150
Salou	Dongsavanh	149	20	18	90
Heng Luang	Dongsavanh	69	13	22	120
Huay Chang	Manchi	92	12	7	30
Keng Chen	Manchi	92	13	23	90
Khouk	Manchi	959	130	15	60
Tha Me	Manchi	631	99	15	60
La Ho	Manchi	176	26	24	120

^*^By motorbike with or without boat

### Questionnaire development

First, we drafted the questionnaire based on the literature [16, 17] and their community-based research experience [7–9, 18–20]. We included question items addressing each of the eight elements of primary health care (health education, food supply and nutrition, safe water and basic sanitation, maternal and child healthcare, immunization, prevention and control of locally endemic diseases, treatment of common diseases and injuries, and provision of essential drugs) [21]. Second, we tested the first version of the questionnaire in two rural villages to ensure the relevance, comprehensiveness, and feasibility of a survey using it. Third, the health center staff tested the second revised version in four villages, and the questionnaire was revised according to the comments from health center staff (Additional file 1: Questionnaire).

### Data collection

The surveyors, who were health center staff, conducted a community-based survey in the 10 villages, using the developed questionnaire. They conducted the survey during a regular outreach activity by interviewing the village health volunteers and observing the environmental conditions of the villages.

There were few village health volunteers in each of the study villages. One village health volunteer per village was invited to be part of the survey. With regard to the questions related to the behavior and knowledge of villagers, village health volunteers were asked to estimate how commonly the villagers practice certain behavior or how knowledgeable on specific health issues. They were asked to choose one from the three response options such as “Most” (i.e., most villagers practice/know), “Half” (i.e., approximately half of villagers practice/know) and “Few/none” (i.e., few/no villagers practice/know).

The observed environmental conditions were the presence of animal feces in the community, and the presence of water containers that mosquito larvae inhabit. The surveyors’ observations were not blinded to village health volunteers, because the target of the observations was the environmental conditions, which village health volunteers would find difficult to improve immediately.

## Results

### Characteristics of participating village health volunteers

All the invited village health volunteers participated in the survey. All were males and aged between 25 and 39 years, with the median age of 30 years. The highest educational attainment of the participants was primary school. The village health volunteers’ working experience varied from 2 to 6 years, with the median of 3 years.

### Common health problems

The common health problems reported in the study villages were diarrhea, followed by malaria, cough, and cold (Table 2). According to estimates, approximately half of the adult villagers did not know how to prevent the health problems. For the reported health problems, village health volunteers conduct health education for their villagers with emphasis on “Three Cleanliness”, which comprised clean food, clean drink, and clean living, as well as immunization and/or the use of a bed net.

**Table 2:** Results of the survey (*n* = 10)

Criteria	Responses	*n*
Part 1: Health education and knowledge about common health problems		
What are common health problems in your village? (multiple answers allowed)	Diarrhea	10
	Malaria	6
	Cough with/without fever	5
	Cold	5
How many adult villagers know how to prevent these health problems?	Diarrhea (*n* = 10, but missing data in the three checklists)	
	Most	0
	Half	7
	Few/none	0
	Malaria (*n* = 6, as the six villages reported malaria to be a problem)	
	Most	0
	Half	5
	Few/none	1
	Cough with/without fever (*n* = 5, as the five villages reported cough to be a problem)	
	Most	1
	Half	4
	Few/none	0
	Cold (*n* = 5, but missing data in the three checklists)	
	Most	0
	Half	2
	Few/none	0
How does the village health volunteer educate villagers about prevention methods?	Emphasizes the importance of Three Cleanliness, immunization, and/or use of bed net	
Part 2: Safe drinking water and basic sanitation		
Where do people get their drinking water?	Pumped well	7
	Rain water	2
	Small-scale water supply	1
Are there any problems with drinking water? If any, describe the problem	Water shortage, unclean water, or need for well	
How many villagers drink boiled water?	Most	2
	Half	4
	Few/none	4
How is garbage disposed of?	Littered	10
	Buried	0
	Burned	0
Is garbage scattered in the village? (Observation)	Yes, much	3
	Yes, but not so much	6
	No	1
Is there any cow/pig feces on the ground? (Observation)	Yes, much	0
	Yes, but not so much	8
	No	2
Are there any latrines in the village?	Yes	0
	No	10
How many households use a latrine?	Not applicable because of no latrines	
Part 3: Food supply and good nutrition		
How many families cannot eat enough food?	Most	3
	Half	3
	Few/none	4
In which months are foods lacking over 1 year?	April and May	3
	January, February, and March	2
	June, July, and August	2
	Other	3
How many mothers give breast milk within 1 h after delivery?	Most	5
	Half	0
	Few/none	5
How many mothers start giving food other than breast milk before the baby is 6 months old?	Most	7
	Half	0
	Few/none	3
How many villagers eat protein-rich foods (e.g., beans, insects, fish, animal meat, eggs) daily?	Most	3
	Half	4
	Few/none	3
How many villagers eat vegetables daily?	Most	3
	Half	2
	Few/none	5
How many villagers eat fruits daily?	Most	1
	Half	6
	Few/none	3
Part 4: Maternal and child health care		
How many adult villagers know how to prevent pregnancy?	Most	1
	Half	6
	Few/none	3
How many families use family planning methods?	Most	1
	Half	4
	Few/none	5
How common is an unwanted pregnancy?	Common	3
	Not so common	2
	Rare	6
How many women deliver at a health center or hospital?	Most	1
	Half	4
	Few/none	5
How many pregnant women receive a prenatal health check-up?	Most	1
	Half	3
	Few/none	6
How many mothers receive a postnatal health check-up?	Most	0
	Half	2
	Few/none	8
Part 5: Immunization		
How many children have been fully vaccinated?	Most	8
	Half	2
	Few/none	0
Part 6: Prevention and treatment of locally endemic diseases		
Are there any water containers, cans, or tires that mosquito larvae inhabit? (Observation)	Yes, many	1
	Yes, but not so many	3
	No	6
How many villagers use a bed net when sleeping in the village?	Most	8
	Half	2
	Few/none	0
How many villagers use a bed net when sleeping in the forest?	Most	1
	Half	5
	Few/none	4
How many villagers wash their hands with soap after defecating?	Most	0
	Half	2
	Few/none	8
How many villagers wash their hands with soap before cooking?	Most	0
	Half	1
	Few/none	9
How many villagers brush their teeth daily?	Most	5
	Half	1
	Few/none	4
How many villagers keep their fingernails clean?	Most	1
	Half	3
	Few/none	6
How many villagers eat raw fish?	Most	4
	Half	3
	Few/none	3
How many adult male villagers smoke tobacco?	Most	6
	Half	3
	Few/none	1
How many adult female villagers smoke tobacco?	Most	0
	Half	0
	Few/none	10
How many households cook inside the house?	Most	0
	Half	5
	Few/none	5
Part 7: Appropriate treatment of common diseases		
How do people treat diarrhea?	Using herbal medicine/using modern medicine/visiting healthcare facility	
How do people treat a fever?	Cooling body with wet towel/visiting healthcare facility/using modern medicine/drinking rice water	
How many villagers use a village health volunteer when necessary?	Most	0
	Half	2
	Few/none	8
How many villagers use a healthcare facility when necessary?	Most	2
	Half	7
	Few/none	1
Part 8: Provision of essential drugs		
Is there a first-aid kit (village medicine bag/box) in the village?	Yes	0
	No	10
Do people have access to essential drugs in the village? If not, where can they get access?	Yes	0
	No	10

### Water and sanitation

The most common water source in the villages was a hand-pumped well, followed by rainwater. Three villages had problems with their water sources (e.g., shortage of water). Only two villages widely practiced the drinking of boiled water. In all 10 villages, villagers disposed of their garbage by littering in various places. Although feces of cows/pigs were observed on the ground in most villages, the amount of feces was not seriously high. No latrines existed in any of the villages.

### Food and nutrition

Most or half of the families were not able to eat enough food in six villages, and the months when such families encountered food shortages tended to differ among the villages. The early initiation of breastfeeding was widely practiced in five villages, but rarely practiced in the other five villages. Exclusive breastfeeding was not widely practiced in most of the villages (*n* = 7). In seven villages, most or half of the families did not eat protein-rich foods daily, including beans, insects, and animal meat. Likewise, most or half of the families did not eat vegetables daily in five villages. In most of the villages (*n* = 9), daily fruit consumption was found to be limited for a number of villagers.

### Maternal and child healthcare

In most of the villages (*n* = 9), half or more of the adult villagers did not know how to prevent pregnancy. Likewise, in most of the villages (*n* = 9), half or more of the families did not use a family planning method. Unwanted pregnancy was common in some villages but not in other villages. In one village, most mothers chose delivery at a healthcare facility or received a prenatal/postnatal health check-up. Most of the children were fully immunized in most of the villages (*n* = 8).

### Prevention and treatment of diseases

Containers with mosquito larvae were found in four villages. In most villages, the majority of villagers used a bed net when sleeping in the village but not when sleeping in the forest. Washing hands with soap before cooking/after defecation was not common in most of the villages. Likewise, brushing teeth daily was not common in the four villages. Eating raw fish was common in some villages but not in others. Smoking tobacco was common in most of the villages, but the behavior was limited to males. Approximately half of the households practiced indoor cooking in five villages. For the treatment of diarrhea, villagers used both herbal and modern medicine. For the treatment of fever, villagers used a method that involved cooling the body with a wet towel, drinking rice water, and modern medicine. In most of the villages (*n* = 8), few villagers used a village health volunteer when necessary. In contrast, in most of the villages (*n* = 9), half or more of the villagers used a healthcare facility when necessary. Essential drugs were not available in the villages, and the villagers had to obtain them at health centers.

## Discussion

The main finding of the present study was that potential risk factors for the reported common health problems were often prevalent in the study villages. The possible risk factors for diarrhea including childhood diarrhea found in the survey were not washing hands with soap, open defecation, not boiling drinking water, not exclusively breastfeeding, the absence of garbage management system and the presence of animal feces on the village ground [22–24]. Likewise, the possible risk factor for malaria found in the survey was not using a bed net when sleeping in the forest [25]. In addition, a possible risk factor for cough was exposure to indoor cooking and tobacco smoke [23].

Most of these risk factors can be addressed without a large financial input. Therefore, with the help of health center staff, villagers can take immediate action. For example, not washing hands with soap can be addressed by making soap available in household handwashing facilities, as handwashing facilities with water are available in approximately 82% of the households in remote rural villages [2]. In addition, not boiling drinking water can be addressed immediately, as boiling water can be performed with locally available resources.

The survey showed that no village implemented a garbage management system. Garbage disposal should be managed, as waste materials such as porcelain and plastic materials can serve as breeding sites of dengue fever vector mosquitoes [26], a disease that is prevalent in the Lao PDR [27].

The survey also showed that villagers were not well informed on how to prevent the reported common health problems, and that village health volunteers (i.e., community health workers in the Lao PDR) educated villagers on limited topics. Community health workers are a cornerstone of primary health care, especially in poor and underserved communities [28, 29]. Therefore, the results of the present study suggest that village health volunteers should be more active by educating villagers on other relevant topics in their villages and by promoting collective actions for village-level issues, such as garbage disposal and latrine construction. A community-based intervention study conducted in the 67 villages of the Phongsaly province, Lao PDR, demonstrated that village facilitators, including village heads and village health volunteers, play a key role in constructing toilets, as well as developing and managing a garbage collection system in their villages [30].

The survey also showed that in more than half of villages, villagers were not able to eat enough food and did not eat protein-rich foods and vegetables daily. Lack of nutrition intake increases vulnerability to diseases, thus leading to various health consequences, including increased mortality and morbidity due to diarrhea, malaria, respiratory illness, and other diseases [24, 31, 32]. To address the shortage of food and poor nutrition intake, intersectoral cooperation with the agricultural sector is critical [33]. The survey also showed that in some villages, villagers were able to eat enough food and ate protein-rich foods and vegetables daily. The survey results suggest that even in a similar environmental setting, an example of village with enough food available was present, and agricultural and food security practices in such example village could be applicable to neighboring villages.

The third most common health problem reported from the study villages was cough. Indoor cooking using biomass fuel (wood) was reported in half of the study villages. Together with tobacco smoke, which was also prevalent among the male adults in the study villages, exposure to cooking smoke is a significant risk factor for respiratory symptoms in the Lao PDR and other low- and middle-income countries [34, 35]. Apart from respiratory symptoms, exposure to the cooking smoke of biomass fuel is also a risk factor for various diseases and health outcomes, including low birth weight and stroke. The health sector should cooperate with other sectors, including the energy and housing sectors, to promote the use of cleaner fuel (e.g., LPG and electricity) and the improvement of housing structure (e.g., ventilation).

Although the present study was confined to villages that share the same background characteristics (i.e., hard-to-reach villages in the same district, with the majority of the residents being of ethnic minorities), there was a wide difference in the seasonality and prevalence of the risk factors among the study villages. For example, the frequency of the daily intake of protein-rich foods, seasons of food insecurity, and level of knowledge of contraception differed greatly among the villages. This between-village heterogeneity within the catchment areas of the same health center is possible, as a malaria risk factor study conducted in the same district also reported significant differences in working and sleeping behaviors, as well as house structure characteristics, between the villages [9]. The between-village heterogeneity observed in the present study suggests that a primary health care intervention should consider the specific local conditions based on the report from the village health volunteers and other key community members.

The Model Healthy Village (MHV) program has been implemented in the Lao PDR since 2007 [36]. A village is certified as an MHV once it meets the evaluation criteria. By 2019, 6679 villages, or 76.7% of the total villages in the country, had been certified as an MHV [37]. Therefore, the MHV program has contributed to ensuring a minimum standard of primary health care in the certified villages of the Lao PDR. However, only eight evaluation criteria are used in the program, while there are 42 evaluation criteria in the questionnaire of the present study. Therefore, the present study was able to capture health-related situations in a comprehensive manner.

The present study has five major limitations. First, because the study was confined to 10 purposively selected villages, the applicability of the study findings to a wider area is of concern. However, we believe that the selected villages are not specific based on the experience of conducting a community-based survey in other remote rural villages of other provinces. Additionally, the study villages are comparable to other remote rural villages of the Savannakhet province, in terms of socio-economic characteristics (i.e., subsistence farming, ethnic minority groups such as Tri and Mangkong, housing, etc.) and ecological characteristics (mountainous area, vegetation, climate, etc.). The findings of the present study are likely to be applicable, at least, to other remote rural villages of Savannakhet province. Second, because the frequency of village members practicing certain behaviors was estimated by the village health volunteers, the accuracy of the estimation remains unknown. However, their estimations seem to be substantially accurate for the following two reasons: first, the surveyors agreed with the estimation results of the village health volunteers. The surveyors are health center staff who frequently visit the study villages for outreach activities and thus they should know the village situations well. Second, some estimation results are comparable to the results of a published study. For example, the majority of the villagers in the present study were estimated to use a bed net in most of the study villages. A household survey-based study conducted in 12 villages in Dongsavanh health center’s catchment area showed that the proportion of people who use a bed net in these villages was 82% [9]. Third, although we ensured the content validity and face validity of the questionnaire through a round of literature review, expert review, and pretest, we did not ensure the construct validity and reliability. Therefore, the quality of some data obtained from the present study might be of concern. As mentioned above, however, some data obtained from the present study are comparable to previous studies, suggesting the possibility that the construct validity is ensured. Fourth, although we used the term “possible risk factor” in the present study, we are unable to determine the causality, as the present study was a cross-sectional study. Finally, the present study did not collect in-depth data from village health volunteers and villagers. Therefore, the present study was unable to explain villagers’ perceptions on and attitudes towards primary health care activities and barriers against promoting recommended health behaviors. Further study using in-depth interview and focus group discussion is recommended to provide a more comprehensive picture of primary health care situations.

## Conclusions

The village health volunteers of the studied villages reported that diarrhea, malaria, and cough were the common health problems. Possible risk factors for these health problems were found in the said villages, and many of these risk factors can be addressed by villagers without a large financial input from outside of the village. However, many villagers were not well informed on how to prevent such health problems. No garbage disposal system was in place, and no latrine was available. Thus, village health volunteers and other key villagers should be more active by educating villagers on the risk factors in their villages and by promoting collective actions for garbage disposal and latrine construction. There is a need for intersectoral actions between the health and other sectors including the agriculture, energy, and housing sectors to address food shortages and indoor air pollution due to indoor cooking using biomass fuel.

## Supplementary Information

**Additional file 1.** Questionnaire.

## Data Availability

The dataset supporting the conclusions of this article is included within the article.
