# Supplementary material for: Primary health care situations in remote rural villages of the Savannakhet province, Lao People’s Democratic Republic
Source: Trop Med Health. 2022 Nov 28;50:90. doi: 10.1186/s41182-022-00482-9 (PMC9703750; doi:10.1186/s41182-022-00482-9)
Supplement: Supplementary file 1 — Additional file 1. Questionnaire. [file 41182_2022_482_MOESM1_ESM.docx]

**Part 1: Health education on health problem and knowledge on its prevention**

| No. | Question | Answer |
| --- | --- | --- |
| 1 | Tell me three common health problems in your village. | 1.  2.  3. |
| 2 | Tell me how to prevent each of these problems. | For the problem 1.    For the problem 2.    For the problem 3. |
| 3 | How many percent of adult villagers know how to prevent each of these health problems? | For the problem 1.  For the problem 2.  For the problem 3. |
| 4 | How does the village health volunteer educate villagers about prevention methods? |  |

**Part 2: Adequate supply of safe drinking water and basic sanitation**

| No. | Question | Answer |
| --- | --- | --- |
| 5 | Where do people get their drinking water?  Multiple-choice | □^1^ Well, □^2^ River,  □^3^Rain water storage,  □^4^ Other ( ) |
| 6 | Are there any problems about drinking water? If any describe the problem. |  |
| 7 | How many villagers do drink boiled water? | □^1^ Most, □^2^ Half,  □^3^ Few/none |
| 8 | Is a well located far from a latrine? |  |
| 9 | How is garbage disposed?  Multiple-choice | □^1^ Just throw away  □^2^ Bury in the ground  □^3^ Burn,  □^4^ Other ( ) |
| 10 | Is garbage scattered in the village? **Observation** | □^1^Yes, many  □^2^ Yes, but not so many  □^3^ No |
| 11 | Are there any feces of cows/pigs on the ground? **Observation** | □^1^Yes, many,  □^2^ Yes, but not so many  □^3^ No. |
| 12 | Are there any latrines in the village? |  |
| 13 | How many households do use a latrine? | □^1^ Most, □^2^ Half,  □^3^ Few/none |

**Part 3: Promotion of food supply and good nutrition**

| No. | Question item | Answer |
| --- | --- | --- |
| 14 | Does the land provide enough food for each family?  Enough? not enough? |  |
| 15 | How many families do have own their land?  For agriculture | □^1^ Most, □^2^ Half,  □^3^ Few/none |
| 16 | How many families cannot eat enough food? | □^1^ Most, □^2^ Half,  □^3^ Few/none |
| 17 | In which months are foods lacking in a year? |  |
| 18 | How many percents of stored rice are damaged by animals including mouse? |  |
| 19 | How many children are too small for their age? | □^1^ Most, □^2^ Half,  □^3^ Few/none |
| 20 | How many children are too skinny? | □^1^ Most, □^2^ Half,  □^3^ Few/none |
| 21 | How many mothers do breastfeed their babies? | □^1^ Most, □^2^ Half,  □^3^ Few/none |
| 22 | How many mothers do give breastmilk within one hour after delivery? |  |
| 23 | How many mothers start giving food other than breast milk before the age of their 6 month? | □^1^ Most, □^2^ Half,  □^3^ Few/none |
| 24 | How many villagers do eat protein-rich foods (example; bean, fish, insect, animal meet, egg) daily? | □^1^ Most, □^2^ Half,  □^3^ Few/none |
| 25 | How many villagers do eat vegetables daily? | □^1^ Most, □^2^ Half,  □^3^ Few/none |
| 26 | How many villagers do eat fruits daily? | □^1^ Most, □^2^ Half,  □^3^ Few/none |

**Part 4: Maternal and child health care**

| No. | Question item | Answer |
| --- | --- | --- |
| 27 | How many adult villagers do know how to prevent pregnancy? | □^1^ Most, □^2^ Half,  □^3^ Few/none |
| 28 | How many families do use family planning methods? | □^1^ Most, □^2^ Half,  □^3^ Few/none |
| 29 | How common is an unwanted pregnancy? | □^1^ Common,  □^2^ Not so common,  □^3^ Rare |
| 30 | How many women do deliver at health center or hospital? | □^1^ Most, □^2^ Half,  □^3^ Few/none |
| 31 | How many pregnant women do receive prenatal health check-up? | □^1^ Most, □^2^ Half,  □^3^ Few/none |
| 32 | How many mothers do receive postnatal health check-up? | □^1^ Most, □^2^ Half,  □^3^ Few/none |
| 33 | How common is a still birth in the village? | □^1^ Common,  □^2^ Not so common,  □^3^ Rare |
| 34 | How many children die before they reach five years of age? | □^1^ Most, □^2^ Half,  □^3^ Few/none |

**Part 5: Immunization**

| No. | Question item | Answer |
| --- | --- | --- |
| 35 | How many children have been vaccinated? | □^1^ Most, □^2^ Half,  □^3^ Few/none |
| 36 | How many children have been fully vaccinated? | □^1^ Most, □^2^ Half,  □^3^ Few/none |

**Part 6: Prevention and treatment of locally endemic diseases**

| No. | Question item | Answer |
| --- | --- | --- |
| 37 | Are there any water containers, cans, bottles, or tires that mosquito larvae inhabit? **Observation** | □^1^Yes, many,  □^2^ Yes, but not so many  □^3^ No |
| 38 | How many villagers do wash their hands with soap after defecating? | □^1^ Most, □^2^ Half,  □^3^ Few/none |
| 39 | How many villagers do wash their hands with soap before cooking? | □^1^ Most, □^2^ Half,  □^3^ Few/none |
| 40 | How many villagers do brush their teeth daily? | □^1^ Most, □^2^ Half,  □^3^ Few/none |
| 41 | How many villagers do take a bath daily? | □^1^ Most, □^2^ Half,  □^3^ Few/none |
| 42 | How many villagers do keep their finger nails clean? | □^1^ Most, □^2^ Half,  □^3^ Few/none |
| 43-1 | How many adult male villagers smoke tobacco? | □^1^ Most, □^2^ Half,  □^3^ Few/none |
| 43-2 | How many adult female villagers smoke tobacco? | □^1^ Most, □^2^ Half,  □^3^ Few/none |
| 44 | How many villages eat raw fish? | □^1^ Most, □^2^ Half,  □^3^ Few/none |
| 45 | How many villagers do drink-drive a motorbike? | □^1^ Most, □^2^ Half,  □^3^ Few/none |
| 46 | How many households do cook inside the house? | □^1^ Most, □^2^ Half,  □^3^ Few/none |
| 47 | How many villagers do wear shoes/sandals? | □^1^ Most, □^2^ Half,  □^3^ Few/none |
| 48 | How many villagers use a bed net when sleeping in the village? | □^1^ Most, □^2^ Half,  □^3^ Few/none |
| 49 | How many villagers use a bed net when sleeping in the forest? | □^1^ Most, □^2^ Half,  □^3^ Few/none |

**Part 7: Appropriate treatment of common diseases and injuries**

| No. | Question item | Answer |
| --- | --- | --- |
| 50 | How do people treat diarrhea? |  |
| 51 | How do people treat a fever? |  |
| 52 | How do people treat injury? |  |
| 53 | How many do villagers use VHV when necessary? | □^1^ Most, □^2^ Half,  □^3^ Few/none |
| 54 | How many do villagers use HC when necessary? | □^1^ Most, □^2^ Half,  □^3^ Few/none |

**Part 8: Provision of essential drugs**

| No. | Question | Answer |
| --- | --- | --- |
| 55 | Is there a first-aid kit (village medicine bag/box) in the village? |  |
| 56 | Do people have access to essential drug in the village? If not, where they can have access? |  |

Name of interviewer (**HC staff**):

Name of respondent (**VHV**):

Village name: Date:
